# Physical Capacity After SARS-CoV-2 Infection in Adolescent Male Soccer Players: A Three-Month Follow-Up Study

**DOI:** 10.7759/cureus.99945

**Published:** 2025-12-23

**Authors:** Andreea-Consuela Timnea-Florescu, Alexandru Dinulescu, Alexandru Cosmin Palcau, Ana Prejmereanu, Olivia Carmen Timnea, Alexandra Floriana Nemes, Roxana Maria Nemes

**Affiliations:** 1 Pediatrics, Titu Maiorescu University, Bucharest, ROU; 2 Pediatrics, Romanian-American University, Bucharest, ROU; 3 Sports Medicine, Chiajna Medical Center, Dudu, ROU; 4 Pediatrics, Carol Davila University of Medicine and Pharmacy, Bucharest, ROU; 5 Pediatrics, Emergency Hospital for Children "Grigore Alexandrescu", Bucharest, ROU; 6 General Surgery, Carol Davila University of Medicine and Pharmacy, Bucharest, ROU; 7 Sports Medicine, Romanian-American University, Bucharest, ROU; 8 Neonatology, Carol Davila University of Medicine and Pharmacy, Bucharest, ROU; 9 Pulmonology, Titu Maiorescu University, Bucharest, ROU; 10 Pulmonology, National Institute of Pneumology Marius Nasta, Bucharest, ROU

**Keywords:** adolescent athletes, covid-19, football, sars-cov-2, soccer

## Abstract

Background and aim

Although many adolescent athletes experience mild or no symptoms following SARS-CoV-2 infection, the potential mid-term effects on physical performance have not been fully characterized. COVID-19 has been associated with alterations in physiological systems related to strength, speed, and aerobic capacity, which may persist after return to sports activity. Furthermore, limited information is available on postinfection recovery trajectories in adolescent athletes, and return-to-play recommendations for this population often rely on data derived from adults. The aim of this study was to evaluate the impact of SARS-CoV-2 infection on physical performance and the recovery trajectory over three months in adolescent male soccer players in Romania.

Methods

A retrospective analysis was conducted on 120 male soccer players aged 12-16 years, equally divided into COVID-19 and non-COVID-19 groups. The COVID-19 group included athletes with confirmed SARS-CoV-2 infection by RT-PCR or rapid antigen testing, while the non-COVID-19 group consisted of athletes with no history of infection, confirmed by negative IgM/IgG serology. Participants underwent weekly fitness tests, including handgrip strength, 10 m and 30 m sprints, bench press, and beep test. For the COVID-19 group, results were analyzed at three time points: one month before infection, one month after infection, and three months postinfection. The Friedman test with Bonferroni correction was applied to assess intragroup changes over time, and differences between the two groups were analyzed using the Mann-Whitney U test. Effect sizes were calculated using Cliff’s delta (δ) for nonparametric comparisons.

Results

All performance metrics showed significant deterioration one month postinfection (p < 0.001). Partial or full recovery was observed at three months. While bench press and 10 m sprint performance returned to baseline levels, handgrip strength, 30 m sprint, and beep test scores remained significantly lower than pre-COVID values. In contrast, the non-COVID-19 group showed stable performance and better results across all time points.

Conclusions

SARS-CoV-2 infection was associated with significant, but predominantly transient, impairments in strength, speed, and aerobic capacity in adolescent soccer players. Although most performance parameters showed partial or complete recovery at three months postinfection, persistent deficits were observed in grip strength and cardiovascular endurance. These results suggest that the neuromuscular and aerobic systems may require longer recovery periods in adolescents compared with other components of performance. Structured functional monitoring and individualized return-to-play protocols are essential to ensure a safe and effective return to sport activity after COVID-19.

## Introduction

Since the onset of the COVID-19 pandemic, caused by SARS-CoV-2, increasing evidence has suggested that even mild or asymptomatic infections can have lasting effects on physiological and psychological functions, including musculoskeletal strength, cardiovascular endurance, and overall physical performance [1-7].

Recent studies have begun to characterize post-COVID impacts in young athletes. Watson et al. (2023) documented quality-of-life changes in more than 17,000 adolescent athletes, while Uluğ et al. (2025) reported performance deficits in elite adolescent athletes practicing upper extremity-dominant sports such as tennis, volleyball, and swimming [8,9]. Research has also demonstrated that athletes who had COVID-19, despite experiencing only mild illness, presented increased respiratory and metabolic demands [10].

Athletic performance encompasses distinct physiological systems, including neuromuscular strength, anaerobic power, and aerobic capacity, each of which may be affected differently by the systemic impact of SARS-CoV-2 [11,12]. Field-based assessments, including handgrip strength, sprint tests, bench press, and beep tests, provide validated, sport-relevant measures of these domains [13-16]. Tracking these parameters longitudinally can reveal differential recovery patterns and inform evidence-based return-to-play protocols [17,18]. In the adult population, the CAESAR studies have provided evidence of COVID-19’s impact on endurance athletes, with significant reductions in cardiopulmonary performance and deteriorations in sleep quality and psychological well-being that indirectly influenced athletic performance and recovery capacity [19,20]. While the CAESAR studies focused on adult endurance athletes, adolescent athletes may experience similar or potentially more pronounced effects due to ongoing physical development and maturation processes, although direct comparisons require careful consideration of physiological differences between age groups.

The application of standardized physical condition assessment protocols is essential for optimizing performance and ensuring safe progression in youth athletes [21]. Regular monitoring of functional domains such as strength, speed, and endurance not only provides benchmarks for training adaptation but also identifies early deficits that may predispose athletes to injury or limit recovery after illness [22,23]. Moreover, there is evidence highlighting sex-specific differences in physical development, physiological responses to exercise, and recovery trajectories in adolescent athletes [24,25]. These differences underline the necessity of establishing tailored assessment and return-to-play protocols for adolescent male and female players, rather than relying on uniform criteria. Integrating such structured evaluations is crucial for advancing evidence-based sports medicine and ensuring long-term athletic development.

This study addresses these limitations by providing a comprehensive, prospective assessment of multiple performance domains in adolescent soccer players over a three-month recovery period. It was hypothesized that all performance parameters would show initial impairment followed by differential recovery patterns, with strength and anaerobic capacity recovering more rapidly than aerobic endurance, and that some deficits would persist compared with uninfected controls at three months postinfection. Accordingly, this study aimed to evaluate changes in strength, speed, and endurance in adolescent soccer players following SARS-CoV-2 infection and to compare their recovery trajectory with that of noninfected controls over a three-month period.

## Materials and methods

Study design

This retrospective cohort study employed a matched-control design to assess changes in physical capacity following SARS-CoV-2 infection. The study was conducted at a single soccer training academy in Romania between January 1, 2021, and December 31, 2021. Participants were identified through a systematic review of academic medical records and training databases. The study utilized existing routine performance assessments that were systematically collected as part of the academy’s standard athlete monitoring program.

Participants

All participants were male soccer players aged 12-16 years, enrolled in the same training academy, and engaged in continuous, organized training for at least six months before study inclusion. Each athlete participated in five 90-minute training sessions and one competitive match per week. Routine weekly performance assessments, including handgrip strength, 10 m and 30 m sprint times, bench press performance, and beep test results, were conducted as part of the academy’s standard monitoring program.

COVID-19 infection in the positive group was confirmed by RT-PCR or rapid antigen testing, while the absence of infection in the control group was verified through negative IgM/IgG serology and lack of infection history. For athletes in the COVID-19 group, data were extracted from the club’s database for three time points: T-1 (one month preinfection), T+1 (first month postinfection), and T+3 (three months postinfection). Because infection dates varied, follow-up intervals were calculated individually from the date of the positive test. COVID-positive players were consecutively enrolled upon meeting inclusion criteria, and control participants were retrospectively matched for age, training status, and measurement timing over an equivalent three-month interval.

Baseline data, including age, height, weight, BMI, training volume, competition level, and COVID-19 symptom severity, were obtained from medical records. All COVID-positive participants were unvaccinated and experienced only mild symptoms (e.g., rhinorrhea, nasal congestion, and occasional cough). COVID-19 symptom severity was obtained retrospectively from medical records and classified as mild based on clinical documentation. A standardized symptom severity scale was not applied at the time of infection.

During isolation, infected athletes followed a structured return-to-training protocol. They completed a period of complete rest during the first three days following diagnosis. Beginning on day four, they performed supervised individual training sessions at home, designed to replicate the team’s usual training content at a reduced intensity (approximately 60-70% of regular training load). These individual sessions were maintained throughout the remainder of the mandatory 10-day isolation period. Athletes rejoined full team training only after completing isolation and resuming normal training intensity.

Exclusion criteria included cardiac involvement or severe COVID-19-related complications (e.g., myocarditis, pericarditis, and multisystem inflammatory syndrome in children), hospitalization exceeding 24 hours, chronic medical conditions that could affect exercise capacity (e.g., uncontrolled asthma, cardiac, neuromuscular, metabolic, endocrine, or rheumatologic disorders), and acute or chronic musculoskeletal injuries within six weeks prior to testing or injuries causing more than seven consecutive days of training absence during any study phase. Additional exclusions included unplanned training load changes greater than 20% from baseline (unless medically indicated), use of medications known to influence performance (e.g., systemic corticosteroids, bronchodilators, and stimulants), and incomplete or undocumented infection or serology data.

Written informed consent was obtained from parents or legal guardians, with assent provided by all participants prior to data collection.

Training program

Throughout the study period, all participants followed the academy’s standardized training program consisting of five 90-minute sessions per week plus one competitive match. Training sessions included technical and tactical drills (30%), physical conditioning (25%), small-sided games (25%), and warm-up and cool-down activities (20%). No major modifications in training content or intensity were implemented during the observation period, aside from the three-day rest taken by COVID-19-positive athletes after diagnosis. Training intensity was standardized at the team level according to the academy’s seasonal training plan and coaching staff supervision. However, objective internal or external load metrics (e.g., heart rate monitoring, GPS data, or session rating of perceived exertion) were not systematically recorded during the study period and therefore could not be included in the analysis.

Physical performance evaluation

All participants underwent weekly standardized performance assessments as part of the academy’s monitoring program, including handgrip strength, 10 m and 30 m sprint times, bench press (chest press), and beep test evaluations. Testing took place at the team’s training facilities, both on the football field and in the gym, under the supervision of the physical trainer, physiotherapist, nurse, and team physician.

A Jamar Hydraulic Hand Dynamometer (JLW Instruments, Chicago, IL, USA) was used to measure handgrip strength. Stopwatches, cones, and electronic timing gates were used for sprint and endurance tests, and a bench with adjustable weights was used for the bench press assessment. Data were compared across three time points: one month preinfection (T-1), first month postinfection (T+1), and three months postinfection (T+3).

Field-based tests were selected for their ecological validity, sensitivity to sport-specific physiological demands, and reproducibility within training environments [26-28].

Hand Grip Strength

Handgrip strength was measured using a calibrated dynamometer following standardized protocols and is a validated indicator of overall neuromuscular function [13].

Sprint Tests (10 m, 30 m)

Sprint performance was assessed using electronic timing gates with high reliability in youth populations (r = 0.94-0.98). The 10 m sprint assessed acceleration, while the 30 m sprint measured sustained sprint performance [15,29].

Bench Press

Upper-body strength was assessed using a standardized progressive load protocol under the supervision of the team’s strength and conditioning staff to ensure safety and validity [30]. Prior to testing, athletes performed a standardized warm-up consisting of five minutes of light aerobic activity followed by dynamic upper-limb mobility exercises. Participants then completed one to two submaximal familiarization sets before attempting the maximal load they could lift with correct technique. Load progression was individualized and based on the athlete’s previous weekly performance records. A rest interval of approximately two minutes was allowed between attempts. The highest successfully completed load was recorded for analysis.

Beep Test

The 20 m multistage shuttle run was used to assess aerobic endurance, which correlates strongly with VO₂max and reflects the intermittent demands of soccer [14,31,32].

Statistical analysis

Data were collected in Microsoft Excel 2016 (Microsoft Corporation, Redmond, WA, USA), and statistical analyses were performed using IBM SPSS Statistics for Windows, Version 26.0 (Released 2018; IBM Corp., Armonk, NY, USA). The Shapiro-Wilk test was used to assess the distribution of quantitative variables. All measured variables were nonnormally distributed (Shapiro-Wilk p < 0.05 for each variable) and are therefore reported as medians with interquartile ranges.

Quantitative variables were compared between independent groups using the Mann-Whitney U test. The Friedman test with Bonferroni correction was used to assess changes in test results across three time points within groups: one month before SARS-CoV-2 infection, one month after infection, and three months after infection.

Effect sizes were calculated using Cliff’s delta (δ) for nonparametric comparisons and computed as follows:

$\delta = \frac{2 \times U}{n_1 \times n_2} - 1,$

where U represents the Mann-Whitney U statistic, and n₁ and n₂ represent the sample sizes of the compared groups. Cliff’s delta thresholds followed established conventions, with values interpreted as negligible (|δ| < 0.147), small (0.147 ≤ |δ| < 0.33), medium (0.33 ≤ |δ| < 0.474), or large (|δ| ≥ 0.474) effect sizes [33].

## Results

Baseline characteristics

The study included 120 adolescent male soccer players (60 per group). The median age was 13.5 years, and the median BMI was 18.4 kg/m². Age (p = 0.177) and BMI (p = 0.571) did not differ significantly between participants with and without SARS-CoV-2 infection, indicating that the groups were well matched (Table 1).

**Table 1:** Characteristics of the two groups

Variable	No SARS-CoV-2 infection group (n = 60)	SARS-CoV-2 infection group (n = 60)	Independent-samples Mann-Whitney U test (p)	Mann-Whitney U statistic
Age (years)	13 (12-14.7)	14 (13-14)	0.177	1552.5
BMI (kg/m²)	18.4 (17.2-19.6)	18.3 (16.7-19.5)	0.571	1908

Recovery trajectory analysis

In the COVID-19 group, performance was assessed at baseline (T-1, preinfection), one month (T+1), and three months postinfection (T+3) (Table 2). All parameters declined at T+1, with partial or full recovery observed by T+3; however, handgrip strength and aerobic endurance (beep test) remained below baseline values.

**Table 2:** Physical capacity changes over time in the COVID-19 group

Parameter	T-1 (preinfection)	T+1 (first month postinfection)	T+3 (three months postinfection)	Friedman test (p) between T-1 and T+1	Friedman χ² statistic between T-1 and T+1	Friedman test (p) between T-1 and T+3	Friedman χ² statistic between T-1 and T+3
Grip strength (kg)	23.5 (20.7-26.6)	18.3 (17.5-25.3)	21.4 (18.9-25.9)	<0.001	60	<0.01	108.21
Bench press (kg)	22.5 (20-25)	20 (15-20)	20 (20-25)	<0.001	47	<0.001	82.85
10 m sprint (s)	2.2 (2.1-2.2)	2.3 (2.2-2.6)	2.3 (2.1-2.5)	0.682	0.76	0.494	1.4
30 m sprint (s)	5.2 (4.9-5.4)	5.4 (5.2-5.7)	5.2 (4.9-5.5)	0.429	1.69	0.318	2.3
Beep test (levels)	8 (7.5-8.5)	7.5 (6.5-8)	8 (7-8)	<0.001	53	0.062	5.63

In the COVID-19 group, parameters showing significant deterioration one month postinfection (p < 0.001) included handgrip strength, which decreased from 23.5 kg to 18.3 kg (a 22% median reduction); bench press capacity, which declined from 22.5 kg to 20.0 kg; and cardiovascular endurance, which decreased from 8.0 to 7.5 beep test levels. Sprint performance did not vary significantly during the study period. At three months postinfection (T+3), handgrip strength and bench press capacity remained significantly lower than preinfection values (p < 0.01 and p < 0.001, respectively). Beep test performance showed a numerical return to baseline median values (8.0 levels); however, this improvement did not reach statistical significance compared with preinfection levels (p = 0.062), indicating incomplete or variable recovery across participants (Table 2).

The control group showed only minor temporal changes. Handgrip strength and bench press performance increased slightly (p < 0.05) over the course of the study period. Sprint performance remained stable, and no significant changes were observed in beep test performance (Table 3).

**Table 3:** Physical capacity changes over time in the non-COVID-19 group

Parameter	T-1	T+1	T+3	Friedman test (p) between T-1 and T+1	Friedman χ² statistic between T-1 and T+1	Friedman test (p) between T-1 and T+3	Friedman χ² statistic between T-1 and T+3
Grip strength (kg)	25 (20.1-28.3)	26.5 (18.5-27.2)	27.3 (23.7-29.3)	0.079	5.06	0.032	6.87
Bench press (kg)	20 (20-25)	20 (20-25)	25 (25-30)	0.087	4.89	0.041	6.4
10 m sprint (s)	2.2 (2.1-2.45)	2.2 (2.1-2.2)	2.1 (2.1-2.3)	0.763	0.54	0.815	0.41
30 m sprint (s)	5 (4.9-5.5)	5.1 (5-5.3)	4.9 (5.2-5.5)	0.494	1.4	0.323	2.26
Beep test (levels)	8 (7.5-8)	8 (7.5-8)	8.5 (8-9)	0.215	3.07	0.563	1.15

Between-group comparisons

At T+1, significant between-group differences emerged across all parameters (Table 4). The COVID-19 group demonstrated substantially inferior performance compared with controls, with medium to large effect sizes: handgrip strength (p < 0.001, δ = 0.48), bench press (p < 0.001, δ = 0.42), sprint performance (10 m: p < 0.001, δ = 0.40; 30 m: p < 0.001, δ = 0.41), and cardiovascular endurance (p < 0.001, δ = 0.38).

**Table 4:** Comparisons between groups at each time point

Parameter	T-1 (preinfection)			T+1 (first month postinfection)			T+3 (three months postinfection)		
	Independent-samples Mann-Whitney U test (p)	Mann-Whitney U statistic	Cliff's Delta (δ)	Independent-samples Mann-Whitney U test (p)	Mann-Whitney U statistic	Cliff's Delta (δ)	Independent-samples Mann-Whitney U test (p)	Mann-Whitney U statistic	Cliff's Delta (δ)
Grip strength	0.092	2120.5	0.17	<0.001	2664.5	0.48	<0.001	2792	0.55
Bench press	0.652	1717.5	-0.04	<0.001	2570	0.42	<0.001	2499.5	0.38
10 m sprint	0.192	2036	-0.13	<0.001	698	0.4	0.007	1299.5	0.27
30 m sprint	0.979	1795	0.003	<0.001	720	0.41	0.013	1333.5	0.23
Beep test	0.108	1502	-0.11	<0.001	2493	0.38	<0.001	2787.5	0.54

At T+3, significant performance gaps persisted between groups (Table 4). The largest differences were observed in handgrip strength (p < 0.001, δ = 0.55) and beep test performance (p < 0.001, δ = 0.54), both demonstrating large effect sizes and indicating clinically meaningful deficits. Sprint performance showed smaller but statistically significant differences (10 m: p = 0.007, δ = 0.27; 30 m: p = 0.013, δ = 0.23), while bench press performance maintained a medium effect size difference (p < 0.001, δ = 0.38).

## Discussion

This study highlights the multidimensional impact of SARS-CoV-2 infection on the physical performance of adolescent male football players by assessing key metrics across strength, speed, and endurance. At one month postinfection, all performance indicators, such as handgrip strength, bench press, 10 m and 30 m sprint times, and beep test scores, were significantly impaired compared with preinfection values. By three months postinfection, most of these deficits showed partial or full recovery, although certain domains, such as handgrip strength and cardiovascular endurance, remained below baseline levels (Table 2).

The most important finding of this study is that SARS-CoV-2 infection in adolescent male soccer players was associated with significant, multidimensional impairments in physical performance one month postinfection, despite a mild clinical presentation and minimal training interruption. Strength, speed, and aerobic endurance all declined substantially at T+1, with medium to large effect sizes, indicating clinically meaningful functional consequences beyond normal training variability.

Although partial recovery was observed by three months postinfection, recovery trajectories differed across performance domains. Anaerobic power and maximal strength parameters (bench press and short sprint performance) demonstrated near-complete recovery, whereas handgrip strength and aerobic endurance showed persistent deficits, both within the COVID-19 group and in comparison with matched controls.

The observed performance decrements cannot be attributed solely to deconditioning, given the minimal training interruption (three days of complete rest followed by individual training). Classical deconditioning studies demonstrate that significant cardiovascular fitness losses require two to four weeks of complete inactivity, with strength losses appearing even later [34,35]. The immediate and substantial deficits observed at one month postinfection (22% reduction in handgrip strength and 9% decline in sprint speed) suggest direct viral pathophysiological effects rather than simple training cessation.

Persistent cardiovascular capacity deficits at three months postinfection may reflect several mechanisms, including direct cardiac effects such as subclinical myocardial involvement [36,37], pulmonary function alterations affecting oxygen uptake [38,39], systemic inflammation impacting mitochondrial function [40,41], or autonomic dysfunction affecting heart rate response [42,43]. The prolonged recovery of aerobic versus anaerobic systems suggests differential physiological impacts that warrant further investigation. These observed physical capacity deficits may affect overall recovery and well-being beyond athletic performance. Reduced physical capacity can limit participation in beneficial physical activity, potentially affecting psychological recovery [44]. However, establishing direct causal relationships between specific physical tests and mental health outcomes requires dedicated assessment tools beyond the scope of this study.

Persistent cardiovascular endurance deficits at three months postinfection may also reflect autonomic nervous system disruption. SARS-CoV-2 can affect vagal function, leading to altered heart rate variability, impaired cardiac output responses to exercise, and reduced parasympathetic recovery capacity [45,46]. This mechanism may explain why aerobic performance, as assessed by the beep test, showed the most persistent deficits, given that aerobic capacity depends heavily on autonomic cardiovascular regulation [47]. Although median beep test performance returned to baseline values by three months postinfection, the lack of statistical significance suggests persistent interindividual variability in endurance recovery. This finding indicates that cardiovascular endurance may recover more slowly or inconsistently than strength parameters in adolescent athletes following SARS-CoV-2 infection.

In this study, Cliff’s delta calculations reveal important practical implications beyond statistical significance (Table 4). At one month postinfection, all performance domains showed medium to large effect sizes (δ = 0.38-0.55), indicating substantial between-group differences. Handgrip strength demonstrated the largest and most persistent effects (δ = 0.48 at one month and δ = 0.55 at three months). The observed median difference was approximately 5 kg (about 20% of baseline performance), which could meaningfully impact sport-specific skills requiring upper-body strength and coordination. Cardiovascular endurance showed a concerning temporal pattern, with effect sizes increasing from medium (δ = 0.38) at one month to large (δ = 0.54) at three months. This widening gap suggests either incomplete recovery in the COVID-19 group or continued improvement in controls. Sprint performance displayed medium effect sizes (δ = 0.40-0.41) at one month, decreasing to small-to-medium effects (δ = 0.23-0.27) at three months. While statistically significant, the median differences of 0.2-0.3 s may fall within normal day-to-day performance variability in adolescent athletes.

These findings have direct implications for post-COVID training protocols in adolescent soccer players. Given that handgrip strength and endurance showed incomplete recovery at three months, rehabilitation programs should emphasize progressive upper-body strengthening (e.g., resistance band and free-weight exercises performed two to three times per week) and aerobic conditioning (e.g., interval running, shuttle runs, and beep test-based drills). In contrast, sprint performance and bench press strength demonstrated near-complete recovery within three months, suggesting that anaerobic and maximal strength components may require shorter reintegration periods. Based on our data, we recommend that athletes recovering from COVID-19 resume low-intensity aerobic training within the first week after symptom resolution, progressively increasing duration and intensity over four to six weeks, while carefully monitoring handgrip strength and endurance capacity. A structured, individualized return-to-play plan should extend beyond the typical two- to four-week reintegration window, with specific attention to restoring neuromuscular and aerobic function.

Our findings align with recent literature indicating that even mild COVID-19 cases in adolescents can lead to functional impairments extending beyond the acute phase [48,49]. Specifically, upper-limb strength, as measured by grip force, remained significantly reduced at three months postinfection, consistent with other studies suggesting possible neuromuscular involvement or lingering inflammatory effects after SARS-CoV-2 infection in youth athletes or nonathletes [9,50,51]. While bench press performance recovered completely by three months, the initial post-COVID-19 decline confirms temporary reductions in maximal muscle output, likely due to deconditioning, reduced training capacity, or systemic fatigue [52]. Speed performance was also affected. Both 10 m and 30 m sprint times increased significantly in the first month post-COVID-19, indicating delayed acceleration and impaired anaerobic performance. By three months, the 10 m sprint had returned to preinfection levels, while the 30 m sprint showed near-complete, but not statistically equivalent, recovery, pointing to a slightly prolonged impact on sustained high-speed efforts [53]. Endurance performance, assessed via the beep test, followed a similar trend, with a significant reduction in the first month postinfection and only partial recovery at three months. These findings mirror other studies reporting decreased aerobic capacity following COVID-19, even in asymptomatic adolescents [54-56]. The slower recovery of cardiovascular endurance compared with speed or strength suggests a differential timeline of physiological restoration, possibly requiring more targeted rehabilitation strategies [57]. Our results are consistent with those of Wezenbeek et al. (2023), who observed persistent aerobic performance deficits in Belgian professional football players for approximately seven weeks postinfection, with elevated %HRmax during Yo-Yo Intermittent Recovery tests at 52.0 ± 11.2 days and full recovery by 127.6 ± 33.1 days [58].

Despite these similarities, several distinctions were noted. The training interruption in our cohort was substantially shorter (three days vs. 12.1 ± 6.1 days in Wezenbeek et al. [58]), yet performance decrements persisted for several months. This finding suggests that viral effects, rather than detraining alone, may underlie prolonged recovery. With respect to recovery timelines, adolescents in our study displayed ongoing handgrip strength and endurance deficits at three months, whereas adult professionals in Wezenbeek et al. demonstrated full recovery of aerobic capacity by approximately four months [58]. These differences likely reflect age-related variations in physiological adaptation and recovery capacity.

Both studies identified aerobic performance as the most affected domain, with anaerobic measures recovering more rapidly. While Wezenbeek et al. found no significant changes in jump, strength, or sprint performance, our data indicated variable recovery patterns across strength and speed domains [58].

The maintenance of individual training during isolation (days 4-10) distinguishes this study from others reporting complete training cessation during quarantine periods. Although athletes remained isolated from group sessions for 10 days, the continuation of structured individual training from day 4 may have mitigated some deconditioning effects, allowing better separation of viral pathophysiological impacts from pure training cessation effects.

Several limitations should be acknowledged when interpreting the findings of this study. The absence of training-matched controls restricts the ability to clearly distinguish COVID-19-specific effects from those associated with brief training interruption, despite the short three-day rest period. Natural developmental variability during adolescence may have masked or exaggerated COVID-related performance changes [59]. Although all participants reported mild symptoms, individual variations in viral load or immune response were not evaluated. The three-month follow-up period may also have been insufficient to fully capture long-term recovery trajectories in this age group. Moreover, while handgrip strength, sprint tests, and the beep test are standardized and reliable assessments in youth athletes [60-62], bench press performance can vary considerably among 12- to 16-year-olds due to differences in technical proficiency, limb proportions, and motor learning development [63,64]. The relatively small sample of players from a single soccer academy limits the generalizability of the findings to broader adolescent athletic populations, other sports, or different training environments. Finally, maturational differences were not assessed because chronological age does not always reflect biological maturity; variations in pubertal stage could have influenced neuromuscular performance outcomes [65,66].

Some methodological aspects may limit precise reproducibility. Although performance testing followed routine academy protocols, certain test-specific parameters (e.g., exact warm-up volume and load progression strategies) were not prospectively standardized or documented. Additionally, training intensity was not quantified using objective load metrics, and COVID-19 symptom severity was assessed clinically rather than using validated scoring instruments. Individual viral load and detailed immunological markers were not available for the COVID-19 cohort, and the follow-up period of three months may not have been sufficient to fully capture long-term recovery trajectories. Day-to-day performance variability inherent to adolescent athletes may also have contributed to fluctuations in test results.

Overall, this study reinforces the importance of individualized return-to-play protocols following COVID-19 infection in adolescents. Given the variation in recovery patterns across strength, speed, and endurance parameters, clinicians, coaches, and physiotherapists should adopt a gradual and tailored approach to training resumption, ensuring sufficient recovery before return to full competitive loads.

## Conclusions

SARS-CoV-2 infection is associated with significant short-term reductions in strength, speed, and endurance in adolescent soccer players, even following mild illness. While most performance parameters improve within three months, handgrip strength and aerobic endurance demonstrate delayed and incomplete recovery. These findings underscore the need for individualized, extended return-to-play monitoring in adolescent athletes following COVID-19. Strength and aerobic capacity should be specifically assessed before full competitive reintegration. Persistent deficits, particularly in aerobic endurance, warrant further investigation into potential underlying physiological mechanisms.
